# Association Between Wearable Device–Based Measures of Physical Frailty and Major Adverse Events Following Lower Extremity Revascularization

**DOI:** 10.1001/jamanetworkopen.2020.20161

**Published:** 2020-11-19

**Authors:** Bijan Najafi, Narek Veranyan, Alejandro Zulbaran-Rojas, Catherine Park, Hung Nguyen, Quinn Kaleikaumaka Nakahara, Hector Elizondo-Adamchik, Jayer Chung, Joseph L. Mills, Miguel Montero-Baker, David G. Armstrong, Vincent Rowe

**Affiliations:** 1 Interdisciplinary Consortium on Advanced Motion Performance, Division of Vascular Surgery and Endovascular Therapy, Michael E. DeBakey Department of Surgery, Baylor College of Medicine, Houston, Texas; 2 Keck School of Medicine, University of Southern California, Los Angeles

## Abstract

**Importance:**

Physical frailty is a key risk factor associated with higher rates of major adverse events (MAEs) after surgery. Assessing physical frailty is often challenging among patients with chronic limb-threatening ischemia (CLTI) who are often unable to perform gait-based assessments because of the presence of plantar wounds.

**Objective:**

To test a frailty meter (FM) that does not rely on gait to determine the risk of occurrence of MAEs after revascularization for patients with CLTI.

**Design, Setting, and Participants:**

This cohort study included 184 consecutively recruited patients with CLTI at 2 tertiary care centers. After 32 individuals were excluded, 152 participants were included in the study. Data collection was conducted between May 2018 and June 2019.

**Exposures:**

Physical frailty measurement within 1 week before limb revascularization and incidence of MAEs for as long as 1 month after surgery.

**Main Outcomes and Measures:**

The FM works by quantifying weakness, slowness, rigidity, and exhaustion during a 20-second repetitive elbow flexion-extension exercise using a wrist-worn sensor. The FM generates a frailty index (FI) ranging from 0 to 1; higher values indicate progressively greater severity of physical frailty.

**Results:**

Of 152 eligible participants (mean [SD] age, 67.0 [11.8] years; 59 [38.8%] women), 119 (78.2%) were unable to perform the gait test, while all could perform the FM test. Overall, 53 (34.9%), 58 (38.1%), and 41 (27.0%) were classified as robust (FI <0.20), prefrail (FI ≥0.20 to <0.35), or frail (FI ≥0.35), respectively. Within 30 days after surgery, 24 (15.7%) developed MAEs, either major adverse cardiovascular events (MACE; 8 [5.2%]) or major adverse limb events (MALE; 16 [10.5%]). Baseline demographic characteristics were not significantly different between frailty groups. In contrast, the FI was approximately 30% higher in the group that developed MAEs (mean [SD] score, 0.36 [0.14]) than those who were MAE free (mean [SD] score, 0.26 [0.13]; *P* = .001), with observed MAE rates of 4 patients (7.5%), 7 patients (12.1%), and 13 patients (31.7%) in the robust, prefrail and frail groups, respectively (*P* = .004). The FI distinguished individuals who developed MACE and MALE from those who were MAE free (MACE: mean [SD] FI score, 0.38 [0.16]; *P* = .03; MALE: mean [SD] FI score, 0.35 [0.13]; *P* = .004) after adjusting by body mass index.

**Conclusions and Relevance:**

In this cohort study, measuring physical frailty using a wrist-worn sensor during a short upper extremity test was a practical method for stratifying the risk of MAEs following revascularization for CLTI when the administration of gait-based tests is often challenging.

## Key Points

**Question:**

Is a wrist-worn frailty meter (FM) that quantifies physical frailty using a 20-second repetitive elbow flexion-extension test a feasible tool for evaluating the risk of 30-day major adverse events (MAEs) after lower-limb revascularization?

**Findings:**

In this 2-center cohort-study of 152 patients, all participants were able to perform the FM test within 1 week before revascularization, whereas most were unable to perform a gait test because of the presence of foot ulcers. The FM distinguished between those with and without 30-day MAEs.

**Meaning:**

The results of this study suggest that the FM is feasible and may support surgeons in evaluating the risks and benefits of revascularization in older adults.

## Teaser

This cohort study tests a frailty meter that does not rely on gait to determine the risk of 30-day major adverse events among patients with chronic limb-threatening ischemia who have undergone lower extremity revascularization.

## Introduction

Frailty is a geriatric syndrome of decreased physiologic reserve and resistance to stressors, which leaves patients more susceptible to poor health outcomes following surgical interventions.^1,2,3,4^ Although recent American College of Surgeons guidelines recommend determining a baseline frailty score for optimal perioperative management of geriatric surgical patients,^5^ such scoring is not routinely performed as part of the preoperative assessment in the hospital setting mainly because of the impracticality and inherent limitations of the current screening tools.

Frailty is often characterized by assessing physical fitness, called physical frailty. Multiple tools have been developed to objectively determine physical frailty.^6,7^ The Fried frailty phenotype has been the criterion standard for clinical assessment of physical frailty.^8^ This method determines physical frailty based on 5 phenotypes, including exhaustion, inactivity, shrinking, slowness, and weakness. The first 3 phenotypes are subjectively assessed with surveys, whereas the last 2 are objectively measured with a grip force test and a 4.5-m walk test. The administration of these tests, specifically the walking test, is challenging in patients with limited mobility, including those with lower extremity peripheral artery disease (PAD) presenting with rest pain, foot ulcers, or amputation.^9^ However, the lack of ability to walk does not necessarily indicate physical frailty and, in addition to incomplete phenotype assessment, compromises the predictive power of the tool.^10,11^

The Frailty Index (FI), proposed by Rockwood et al,^12^ is another validated tool that is based on deficit accumulation. However, because it is not representative of physical frailty, it has limitations for informing or tracking potential benefits of preventive solutions to improve resilience and physical fitness among frail patients, such as prerehabilitation before surgery.^7^

To address the limitations of the Fried criteria and the Rockwood FI, we proposed an alternative tool for measuring physical frailty using a wrist-worn sensor called the frailty meter (FM). The FM quantifies physical frailty phenotypes such as weakness, slowness, range of motion (rigidity), and exhaustion, measured during a 20-second repetitive elbow flexion-extension task, into a continuous scale ranging from 0 to 1 using an optimized linear regression model described by Lee et al.^9^ This model was developed based on previous studies, which showed a strong correlation between the FM FI and the modified Rockwood FI and estimated unfavorable hospital discharge among geriatric trauma patients.^1,13,14^ The FM facilitates measuring physical frailty in both ambulatory and nonambulatory patients.^15,16,17^

We tested the feasibility of using FM in the inpatient setting as well as its ability to determine the risk of occurrence of major adverse events (MAEs) during the first 30 postoperative days in patients with PAD undergoing lower extremity revascularization for chronic limb-threatening ischemia (CLTI). Our hypotheses were as follows: (1) FM is a feasible tool for assessing frailty in patients with CLTI in the inpatient setting and (2) the preoperative FI, generated by FM, enables distinguishing revascularized patients with and without occurrence of MAEs during the first 30 postoperative days.

## Methods

### Study Design and Setting

A consecutive sample of 184 patients with PAD who underwent revascularization with open bypass surgery (OBPS) or endovascular therapy (EVT) at St Luke’s Medical Center of Baylor College of Medicine (BCM; Houston, Texas) from September 2016 to May 2018 and at 2 teaching hospitals of University of Southern California (USC; Keck Medical Center of USC and Los Angeles County–USC Medical Center, Los Angeles) from September 2018 to June 2019 were recruited into the study. All surgeons at BCM site (M.M., J.C, and J.M) were masked to the outcomes of the FM test. The surgeon at USC site (V.R.) was not masked, but the decision for revascularization was not based on the outcome of the test.

### Participants

The inclusion and exclusion criteria were selected according to previously reported data,^18,19,20,21,22^ which included any adult patient undergoing lower extremity revascularization for PAD. Patients were excluded if they presented with acute limb ischemia; were unable to fully comply with the follow-up protocol (eg, long-distance travel) or to provide informed consent; had any acute illness such as myocardial infarction or stroke; had nonatherosclerotic vascular disease; or had undergone any major surgery within the preceding 30 days. Patients with claudication (ie, Rutherford score <4)^23^ were excluded because of the different risk profiles and treatment strategies compared with CLTI (Rutherford score ≥4). Rutherford classes were used because wound, ischemia, foot infection (WIfI) stratification^24^ was not uniformly done at the time of the study in all enrolling institutions. Participants were provided written informed consent, approved by the institutional review boards of BCM or USC. This study followed the Strengthening the Reporting of Observational Studies in Epidemiology (STROBE) reporting guideline.

### Measurement Procedure

**Figure 1.**
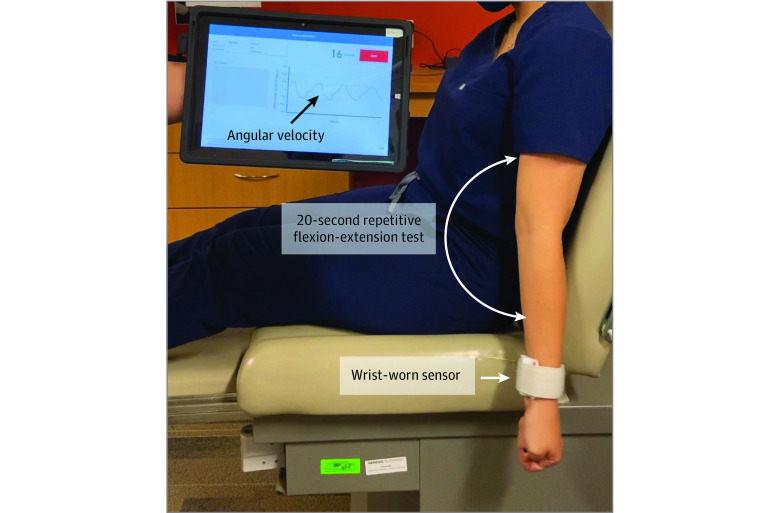
Frailty Assessment by Upper Extremity Motion Using Frailty Meter The system, which consists of a sensor worn on the wrist and a wirelessly connected tablet, records angular velocity during a 20-second repetitive elbow flexion-extension task, and then displays a frailty score. All measurements were done in the clinics or in the preprocedure holding area of the catheterization laboratory within 1 week before the revascularization.

The FM test was performed within 1 week before the revascularization procedure. The FM consists of 1 wearable sensor and a wirelessly connected tablet (Figure 1). It records the angular velocity during a 20-second repetitive elbow flexion-extension task and then displays an FI. Participants were tested on their dominant arm, similar to the previously established protocol.^9,16^

Participants were classified, based on their FI, as robust (FI <0.20), prefrail (FI ≥0.20 to <0.35), or frail (FI ≥0.35) using the benchmark proposed by Rockwood et al^25^ and the density function suggested by Li et al.^20^ Research coordinators (N.V. at USC and A.Z. at BCM) reviewed institutional electronic medical records for patient medical history and demographic data including ethnicity to differentiate whether MAE occurrence was associated with any race. The initial protocol also included assessing physical frailty using the Fried frailty phenotype criteria.^19^ However, after recruiting the first 10 patients, it became evident that most participants were unable to perform a gait test and/or answer the physical activity questionnaire, 2 important phenotypes of the Fried criteria. We therefore decided to remove this test from the protocol.

### Definition of MAEs

MAEs were defined according to the criteria proposed by Conte et al^18^ and were further subcategorized as major adverse cardiovascular events (MACEs) and major adverse limb events (MALEs). MACEs included myocardial infarction, stroke, and a death from any cause. MALEs included unplanned major amputations or major reinterventions (eg, thrombectomy, thrombolysis, or bypass graft placement). These MAEs were documented up to 30 days following the procedure.

### Statistical Analysis

All continuous data are presented as means and SDs. All categorical data are expressed as counts and percentages. One-way analysis of variance for normally distributed variables or Kruskal-Wallis H test for nonnormally distributed variables were used to estimate differences of means between frailty groups (ie, robust, prefrail, and frail), followed by Games-Howell post hoc test for pairwise analysis. The Mann-Whitney *U* test was used to determine significant difference between groups with and without MAEs. If a categorical variable had more than 2 levels, the χ^2^ test was used to determine significant level. The effect size was measured using odds ratios (ORs) and 95% CIs in case of categorical variables and Cohen *d* for continuous variables. Generalized estimating equations were conducted to assess the association between FI and either frailty or MAE status by adjusting continuous variables proven to be associated with frailty.^26,27,28^ Statistical significance was set at a 2-sided *P* < .05. All statistical analyses were performed using SPSS statistical software version 24 (IBM) and MATLAB version R2018b (MathWorks).

## Results

The study initially recruited 184 potentially eligible patients; however, 18 (9.7%) were subsequently excluded for presenting with claudication alone, 8 (4.3%) because of FM technical failure (lack of communication between sensor and tablet or not saving data after test), and 3 (1.6%) were lost to follow-up. Three participants (1.6%) were excluded after consent and enrollment, including 2 patients because of cancellation of the procedure and 1 because of severe cognitive impairment and the inability to undergo the full protocol. The remaining 152 eligible patients with CLTI were monitored for 30 days after surgery.

### Clinical and Demographic Characteristics

Participants had a mean (SD) age of 67.8 (11.8) years and mean (SD) body mass index (BMI; calculated as weight in kilograms divided by height in meters squared) of 27.6 (6.3). Overall, 59 women (38.8%) were in the sample. The median (range) Rutherford score was 5 (4-6). PAD risk factors, such as diabetes (64 of 75 [85.3%]), hypertension (64 of 152 [42.1%]), and history of tobacco use (92 of 152 [61.3%]) were highly prevalent. In addition, 119 patients (78.2%) had a foot ulcer at the time of assessment, and thus, the walking test was discouraged. In this cohort, 115 patients (75.6%) underwent EVT (eg, atherectomy, balloon or stent angioplasty), while 37 (24.3%) underwent OBPS. The mean (SD) hospital length of stay was 11.8 (18.1) days including both inpatient and outpatient settings. Characteristics and specific sites of anatomic lesions were not included in this study.

### Robust, Prefrail, and Frail Groups

**Figure 2.**
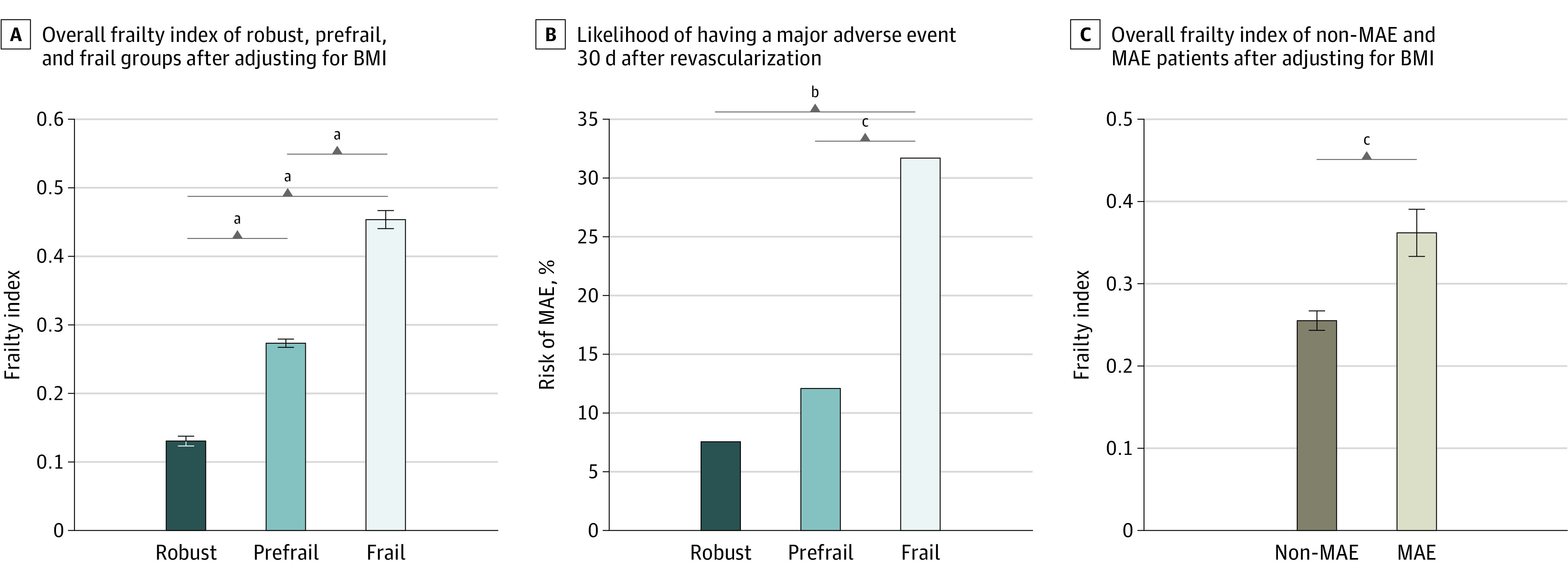
Overall Frailty Index and Risk of Major Adverse Events (MAEs) A and C, Whiskers indicate standard errors. BMI indicates body mass index. ^a^*P* ≤ .001. ^b^*P* ≤ .01. ^c^*P* < .05.

Based on the FM test, 53 participants (34.9%) were classified as robust, 58 (38.1%) as prefrail, and 41 (27%) as frail (Table 1 and Figure 2A). Mean (SD) age was significantly different among the robust (65.2 [11.5] years), prefrail (67.2 [11.8] years) and frail (71.9 [11.2] years) groups (*P* = .02). Sex, height, weight, and BMI did not differ between groups. The mean (SD) FI was not significantly different between patients with open (0.26 [0.13]) and endovascular (0.28 [0.14]) revascularizations.

**Table 1.** Demographic Characteristics, Frail Meter Parameters, and Risk Factors of Participants Undergoing Revascularization

Characteristic	Patients, No./total No. (%), by group			*P* value
	Robust (n = 53)	Prefrail (n = 58)	Frail (n = 41)	
Demographic characteristics				
Age, mean (SD), y	65.2 (11.5)	67.2 (11.8)	71.9 (11.2)	.02
Women	17/53 (32.1)	22/57 (38.6)	20/41 (48.8)	.26
Height, mean (SD), cm	165 (9)	167 (18)	165 (11)	.40
Weight, mean (SD), kg	73.7 (13.3)	83.7 (27.1)	72.7 (19.7)	.14
BMI, mean (SD)	27.3 (5.2)	28.7 (7.0)	26.5 (6.4)	.47
Mobility aid	7/13 (53.8)	22/35 (62.9)	21/26 (80.8)	.17
Race/ethnicity				
White	18/53 (34.0)	23/57 (40.4)	14/41 (34.1)	.26
African American	6/53 (11.3)	11/57 (19.3)	10/41 (24.4)	
Hispanic or Latino	29/53 (54.7)	22/57 (38.6)	17/41 (41.5)	
Asian	0	1/57 (1.8)	0	
Peripheral artery disease risk factors				
History of foot ulcer	8/13 (61.5)	29/36 (80.6)	17/26 (65.4)	.28
Diabetes	11/13 (84.6)	31/36 (86.1)	22/26 (84.6)	.98
Hypertension	33/53 (62.3)	19/58 (32.8)	12/41 (29.3)	.001
Heart disease	17/53 (32.1)	25/58 (43.1)	15/41 (36.6)	.48
Stroke	3/53 (5.7)	7/58 (12.1)	10/41 (24.4)	.03
Renal disease	18/53 (34)	11/58 (19)	9/41 (22)	.17
History of tobacco use	28/53 (52.8)	36/56 (64.3)	28/41 (68.3)	.27
Prescription medications, mean (SD), No.	7.9 (4.0)	8.8 (4.2)	8.9 (4.1)	.75
History of fall	3/11 (27.3)	6/30 (20.0)	12/23 (52.2)	.04
Comorbidities, mean (SD), No.	3.2 (1.7)	2.8 (2.9)	3.0 (1.5)	.57
Rutherford score, mean (SD)	5.3 (0.8)	5.3 (1.5)	5.2 (0.9)	.97
Length of hospital stay, mean (SD), d	10.92 (7.75)	9.65 (16.52)	16.32 (20.41)	.20
Type of intervention				
Open	13/53 (24.5)	16/58 (27.6)	8/41 (19.5)	.65
Endovascular	40/53 (75.4)	42/58 (72.4)	33/41 (80.5)	
Frail meter parameters and phenotypes, mean (SD)^a^				
Overall frailty index score	0.13 (0.05)	0.27 (0.04)	0.45 (0.08)	<.001
Speed, ie, slowness, deg/s	1101 (364)	665 (210)	327 (176)	<.001
Speed reduction, ie, exhaustion, %	10.74 (24.36)	−3.38 (12.08)	−15.03 (20.87)	<.001
ROM, ie, flexibility, deg	107 (33)	80 (27)	40 (24)	<.001
Power, ie, weakness, deg^2^/s^3^ x 10^5^	2.63 (1.91)	0.84 (0.75)	0.23 (0.33)	<.001
Flexion time, ms	336 (77)	425 (133)	600 (360)	<.001
Flexions, No.	30 (8)	24 (8)	20 (9)	<.001

Abbreviations: BMI, body mass index (calculated as weight in kilograms divided by height in meters squared); ROM, range of motion.

^a^ Associated Fried phenotypes were determined according to the study by Toosizadeh et al.^16^

Rates of diabetes, history of tobacco use, Rutherford score, length of hospital stay, total number of prescription medications, and total number of comorbidities demonstrated no significant differences among groups. However, hypertension, stroke history, and history of fall were significantly different between groups (hypertension: robust, 33 [62.3%]; prefrail, 19 [32.8%]; frail, 12 [29.3%]; *P* = .001; history of stroke: robust, 3 [5.7%]; prefrail, 7 [12.1%]; frail, 10 [24.4%]; *P* = .03; history of fall: robust, 3 of 11 [27.3%]; prefrail, 6 of 30 [20.0%]; frail, 12 of 23 [52.2%]; *P* = .04).

During the 30-day postoperative period, MAEs occurred in 13 patients in the frail group (31.7%), 7 patients in the prefrail group (12.1%), and 4 patients in the robust group (7.5%; *P* = .004) (Table 2, Figure 2B). The frail group developed MAEs 2.1 times more frequently than the robust group (OR, 5.69; 95% CI, 1.69-19.13; *P* = .003), and 1.8 times more frequently than the prefrail group (OR, 3.38; 95% CI, 1.21-9.46; *P* = .02).

**Table 2.** Comparison of MAEs Among the 3 Groups

Outcome	Groups, No. (%)			
	Robust (n = 53)	Prefrail (n = 58)	Frail (n = 41)	*P* value
MACE	1 (1.9)	3 (5.2)	4 (9.8)	.24
MALE	3 (5.7)	4 (6.9)	9 (22.0)	.02
Total MAE	4 (7.5)	7 (12.1)	13 (31.7)	.004

Abbreviations: MACE, major adverse cardiovascular event; MAE, major adverse event; MALE, major adverse limb event.

The rate of open surgeries was higher in the prefrail group (robust, 13 [24.5%]; prefrail, 16 [27.6%]; frail, 8 [19.5%]), while the rate of endovascular procedures was higher in the frail group (robust, 40 [75.4%]; prefrail, 42 [72.4%]; frail, 33 [80.5%]). However, there was no significant difference for type of surgery among groups (*P* = .65).

### MAEs, MACEs, and MALEs

Of the 152 participants who were observed for 30 days after surgery, 24 (15.7%) developed MAEs (Table 3). Except BMI, none of the demographic factors were statistically significantly different between the MAE and non-MAE groups. Common PAD risk factors, such as history of foot ulcer, diabetes, hypertension, heart disease , stroke, and history of tobacco use, also showed no significant differences between these 2 groups. The MAE group had a relatively higher rate of history of tobacco use (OR, 2.10; 95% CI, 0.78-5.67; *P* = .13), but the differences failed to achieve a statistical significance in our sample. The MAE rate during the postoperative 30 days was not different between patients who underwent an open (7 of 37 [18.9%]) or endovascular (17 of 115 [14.7%]) revascularization. Overall, the frail group had the most deaths (3 of 4 [75.0%]) and limb loss (5 of 9 [55.5%]) reported.

**Table 3.** Demographic Characteristics, Risk Factors, and Frail Meter Parameter of Patients With and Without MAEs

Characteristic	No./total No. (%), by group		*P* value	OR (95% CI)
	Non-MAE (n = 128)	MAE (n = 24)		
Demographic characteristics				
Age, mean (SD), y	67.4 (11.7)	70.2 (12.0)	.30	NA
Women	51/127 (40.2)	8/24 (33.3)	.53	0.74 (0.29 to 1.86)
Height, mean (SD), cm	165 (14)	168 (11)	.57	NA
Weight, mean (SD), kg	78.1 (21.7)	72.8 (20.3)	.23	NA
BMI, mean (SD)	28.0 (6.2)	25.7 (6.6)	.04	NA
Use of mobility aid	44/65 (67.7)	6/9 (66.7)	.95	0.95 (0.21-4.19)
Race/ethnicity				
White	41/127 (32.3)	14/24 (58.3)	.01	NA
African American	24/127 (18.9)	3/24 (12.5)	.46	NA
Hispanic or Latino	61/127 (48.0)	7/24 (29.2)	.09	NA
Asian	1/127 (0.8)	0	.19	NA
Peripheral artery disease risk factor				
History of foot ulcer	48/66 (72.7)	6/9 (66.7)	.70	0.75 (0.16-3.32)
Diabetes	56/66 (84.8)	8/9 (88.9)	.75	1.42 (0.16-12.70)
Hypertension	53/128 (41.4)	11/24 (45.8)	.69	1.19 (0.49-2.87)
Stroke	18/128 (14.1)	2/24 (8.3)	.45	0.55 (0.12-2.56)
Heart disease	48/128 (37.5)	6/24 (25.0)	.24	0.55 (0.20-1.49)
Renal disease	32/128 (25.0)	6/24 (25.0)	>.99	1.00 (0.36-2.73)
History of tobacco use	74/126 (58.7)	18/24 (75.0)	.13	2.10 (0.78-5.67)
Prescription medication, mean (SD), No.	8.7 (4.1)	8.4 (4.4)	.89	NA
History of fall	17/55 (30.9)	4/9 (44.4)	.42	1.78 (0.42-7.50)
Comorbidities, mean (SD), No.	3.0 (1.6)	3.0 (1.4)	.73	NA
Rutherford score, mean (SD)	5.3 (0.8)	5.4 (0.8)	.48	NA
Frail meter parameters and phenotypes, mean (SD)^a^				
Frailty index	0.26 (0.13)	0.36 (0.14)	.001	NA
Speed, ie, slowness, deg/s	755 (412)	592 (379)	.08	NA
Speed reduction, ie, exhaustion, %	1.0 (21.7)	−13.2 (21.1)	.02	NA
ROM, ie, flexibility, deg	82 (38)	62 (42)	.02	NA
Power, ie, weakness deg^2^/s^3^ × 10^5^	1.38 (1.63)	0.99 (1.43)	.13	NA
Flexion time, ms	423 (198)	536 (367)	.26	NA
Flexions, No.	25 (9)	23 (9)	.62	NA

Abbreviations: BMI, body mass index (calculated as weight in kilograms divided by height in meters squared); MAE, major adverse event; NA, not applicable; OR, odds ratio; ROM, range of motion.

^a^ Associated Fried phenotypes were determined according to the study by Toosizadeh et al.^16^

Analysis of FM parameters demonstrated that the mean (SD) FI was higher in the MAE group compared with the non-MAE group (0.36 [0.14] vs 0.26 [0.13]; *P* = .001; *d* = 0.76). Except for mean [SD] scores on speed reduction (ie, exhaustion; mean [SD], −13.2% [21.1%] vs 1.0% [21.7%]; *P* = .02; *d* = 0.66) and flexibility (62° [42°] vs 82° [38°]; *P* = .02; *d* = 0.52), the remainder of the measured metrics, while worse in the MAE group than in the non-MAE group, did not reach statistical significance.

After adjusting for BMI, the mean (SD) FI score was still found to be significantly higher in the MAE than in non-MAE group (0.36 [0.14] vs 0.26 [0.13]; *P* = .001) (Figure 2C). Patients who experienced MAEs were further subdivided into MACE (n = 8) and MALE (n = 16) groups to conduct a pairwise comparison for the FI after adjusting for BMI. The results revealed a significantly lower mean (SE) FI in non-MAE (0.25 [0.13]) compared with MACE (0.38 [0.16]; *P* = .03) and MALE (0.35 [0.13]; *P* = .004) groups, but there was no significant difference of FI between MACE and MALE groups.

## Discussion

Assessment of physical frailty is important for comprehensive perioperative care, especially in the geriatric population. However, a quick, accurate, and simple tool to assess physical frailty is not widely available yet. This issue is particularly challenging for patients with CLTI, who frequently present with ischemic rest pain, foot ulcers, or lower extremity amputation and are thus often unable to perform the gait-based assessments that are part of current well-established physical frailty assessment tools. In this study, we proposed the use of an alternative digital tool to assess physical frailty during a 20-second repetitive elbow flexion-extension test using a wrist-worn sensor called FM. All participants in this cohort study were able to perform the FM test, indicating high feasibility. In contrast, 119 patients (78.2%) were unable to perform gait-based protocols because of the presence of chronic ulcers or tissue loss. The preoperative sensor-derived FI could allow for the identification of patients with CLTI who are more likely to experience postoperative MAEs, supporting the potential ability of FM to estimate the risk of MAEs after lower extremity revascularization.

More recently, to integrate frailty screening as a component of routine preoperative assessment, the focus has shifted toward developing a quick and objective assessment tool to evaluate physical frailty based on a single frailty phenotype. Grip strength, which is often associated with sarcopenia (weight loss phenotype) and reduced muscle strength (weakness phenotype), has been shown to be a robust surrogate measure of frailty,^21,22^ predicting disability, morbidity, and mortality^22,29,30^ as well as high cardiovascular risk^31^ in geriatric patients. However, in the study by Dudzinska-Griszek et al,^32^ 30% of patients were unable to fulfil the frailty criterion of weakness, suggesting that it can be difficult to measure grip strength on a frail hospitalized patient. Another study by Theou et al,^11^ which examined more than 27 000 participants, found that 9.7% of the participants were missing a grip strength score and 61.1% of participants were unable to perform the test. In our study, of all measured physical frailty phenotypes, exhaustion and flexibility were the significant parameters able to distinguish MAE with medium effect sizes, comparable with the large effect size observed for FI. This indicates that both exhaustion and weakness are important frailty phenotypes in distinguishing MAEs, although measuring the other mentioned phenotypes may improve the capacity of distinguishing MAE.

Based on current literature, the type of intervention could also be associated with the risk of postoperative adverse events following lower extremity revascularization for PAD. Results of the randomized controlled BASIL study showed that there were no differences in the early, overall mortality rates between OBPS and EVT, but OBPS was associated with increased early morbidity.^33^ Conversely, Mehaffey et al^34^ showed that, compared with EVT, lower extremity OBPS resulted in lower rates of MALEs but similar rates of MACEs. In our study, there was no significant difference of MAE rates between patients who received OBPS and those who received EVT. The mean (SD) FI also was not significantly different between patients with open (0.26 [0.13]) and endovascular (0.28 [0.14]) revascularizations. Previous studies have shown that patients with CLTI have higher mortality^35^ and increased risk of an amputation following a revascularization^36,37^ especially in frail patients.^38^ In our study, 4 patients (2.6%) died and 9 patients (5.9%) had limb loss within 30 days of revascularization. Interestingly, 3 of these deaths as well as 5 of these amputations belonged to patients in the frail group, suggesting that higher FI scores might be able to distinguish worse outcomes after revascularization.

Another important observation is the association of FM with MAEs regardless of the type of intervention.^39^ For instance, even a minor intervention (eg, balloon angioplasty) could have fatal consequences for a patient at high risk of morbidity or mortality (ie, considered frail).^26^ Although MACEs have been associated with supra-inguinal lesions,^24^ in our study, all frail patients who died (n = 3) within 30 days of revascularization underwent infra-inguinal percutaneous angioplasties. Thus, the FM may be a patient-risk assessment tool useful for estimating the likelihood of MAE, regardless of the anatomic site of the lesion treated. On the other hand, the FM might be able to distinguish whether robust patients could tolerate high-risk interventions (eg, OBPS) without risk of postintervention MAEs. In this study, 13 patients (24.5%) from the robust group underwent open interventions, and only 1 patient developed an MACE (nonmortal event) and 1 patient developed an MALE (non–limb loss event), suggesting that FI could be a predictive tool independent of the type of surgery.

A demographic shift of geriatric surgical patients has spurred the need for a routine assessment tool to accurately distinguish postoperative outcomes.^40^ In this study, age as well as the number of comorbidities were not significantly different between the MAE and non-MAE groups. However, the likelihood of an MAE increased with the increase in the sensor-derived FI score. The prefrail group had 1.3 times higher risk of developing an MAE compared with the robust group, although this difference was statistically nonsignificant. The frail group developed MAEs 2.1 times more frequently than the robust group and 1.3 times more frequently than the prefrail group. Furthermore, patients who experienced an MACE had the highest FI score.

### Limitations

This study has limitations. It did not focus on the specific type of intervention for EVT (eg, scaffolding, balloon angioplasty, or atherectomy), OBPS (eg, autologous, prosthetic), or vascular lesion anatomy. Similarly, MACE and MALE breakdown were not reported. The primary outcome was to test the feasibility of the FM regardless of specific MAE or type of intervention. Thus, we generalized both outcomes to report homogeneous MAEs. This study was not designed to estimate the rate of MAEs and thus may be underpowered for estimating MAEs after lower extremity revascularization. Future studies to explore the association of revascularization with reversing frailty status are warranted.

## Conclusions

In this cohort study, a wrist-worn sensor and a 20-second repetitive elbow flexion-extension exercise were feasible for determining preoperative physical frailty and its ability to distinguish between those with and without postoperative MAEs in patients with PAD undergoing open or endovascular revascularization. This quick and low-risk test could facilitate the integration of physical frailty measurement into routine preoperative patient assessment and supports surgeons’ decision-making in evaluating the ratio of risk from revascularization in older adults.

## References

[1] Joseph B, Pandit V, Zangbar B, Validating trauma-specific frailty index for geriatric trauma patients: a prospective analysis. J Am Coll Surg. 2014;219(1):10-17.e1. doi:10.1016/j.jamcollsurg.2014.03.020 24952434

[2] Makary MA, Segev DL, Pronovost PJ, Frailty as a predictor of surgical outcomes in older patients. J Am Coll Surg. 2010;210(6):901-908. doi:10.1016/j.jamcollsurg.2010.01.028 20510798

[3] Shinall MC Jr, Arya S, Youk A, Association of preoperative patient frailty and operative stress with postoperative mortality. JAMA Surg. 2019;155(1):e194620. doi:10.1001/jamasurg.2019.4620 31721994 PMC6865246

[4] Winograd CH, Gerety MB, Chung M, Goldstein MK, Dominguez F Jr, Vallone R Screening for frailty: criteria and predictors of outcomes. J Am Geriatr Soc. 1991;39(8):778-784. doi:10.1111/j.1532-5415.1991.tb02700.x 1906492

[5] Chow WB, Rosenthal RA, Merkow RP, Ko CY, Esnaola NF; American College of Surgeons National Surgical Quality Improvement Program; American Geriatrics Society Optimal preoperative assessment of the geriatric surgical patient: a best practices guideline from the American College of Surgeons National Surgical Quality Improvement Program and the American Geriatrics Society. J Am Coll Surg. 2012;215(4):453-466. doi:10.1016/j.jamcollsurg.2012.06.017 22917646

[6] Mohler MJ, Fain MJ, Wertheimer AM, Najafi B, Nikolich-Žugich J The frailty syndrome: clinical measurements and basic underpinnings in humans and animals. Exp Gerontol. 2014;54:6-13. doi:10.1016/j.exger.2014.01.024 24503059

[7] Cesari M, Gambassi G, van Kan GA, Vellas B The frailty phenotype and the frailty index: different instruments for different purposes. Age Ageing. 2014;43(1):10-12. doi:10.1093/ageing/aft160 24132852

[8] Fried L, Walston J Frailty and failure to thrive Hazzard WR, Blass JP, Ettinger WH, Jr, Halter JB, Ouslander J, eds. Principles of Geriatric Medicine and Gerontology. Fourth Edition. McGraw Hill; 1998:1387-1402.

[9] Lee H, Joseph B, Enriquez A, Najafi B Toward using a smartwatch to monitor frailty in a hospital setting: using a single wrist-wearable sensor to assess frailty in bedbound inpatients. Gerontology. 2018;64(4):389-400. doi:10.1159/000484241 29176316 PMC5970017

[10] Theou O, Cann L, Blodgett J, Wallace LM, Brothers TD, Rockwood K Modifications to the frailty phenotype criteria: systematic review of the current literature and investigation of 262 frailty phenotypes in the Survey of Health, Ageing, and Retirement in Europe. Ageing Res Rev. 2015;21:78-94. doi:10.1016/j.arr.2015.04.001 25846451

[11] Theou O, Brothers TD, Mitnitski A, Rockwood K Operationalization of frailty using eight commonly used scales and comparison of their ability to predict all-cause mortality. J Am Geriatr Soc. 2013;61(9):1537-1551. doi:10.1111/jgs.12420 24028357

[12] Rockwood K, Song X, MacKnight C, A global clinical measure of fitness and frailty in elderly people. CMAJ. 2005;173(5):489-495. doi:10.1503/cmaj.050051 16129869 PMC1188185

[13] Joseph B, Pandit V, Rhee P, Predicting hospital discharge disposition in geriatric trauma patients: is frailty the answer? J Trauma Acute Care Surg. 2014;76(1):196-200. doi:10.1097/TA.0b013e3182a833ac 24368379

[14] Toosizadeh N, Najafi B, Reiman EM, Upper-extremity dual-task function: an innovative method to assess cognitive impairment in older adults. Front Aging Neurosci. 2016;8:167. doi:10.3389/fnagi.2016.00167 27458374 PMC4935727

[15] Toosizadeh N, Joseph B, Heusser MR, Assessing upper-extremity motion: an innovative, objective method to identify frailty in older bed-bound trauma patients. J Am Coll Surg. 2016;223(2):240-248. doi:10.1016/j.jamcollsurg.2016.03.030 27155751 PMC4961594

[16] Toosizadeh N, Mohler J, Najafi B Assessing upper extremity motion: an innovative method to identify frailty. J Am Geriatr Soc. 2015;63(6):1181-1186. doi:10.1111/jgs.13451 26096391 PMC6653678

[17] Joseph B, Toosizadeh N, Orouji Jokar T, Heusser MR, Mohler J, Najafi B Upper-extremity function predicts adverse health outcomes among older adults hospitalized for ground-level falls. Gerontology. 2017;63(4):299-307. doi:10.1159/000453593 27941328 PMC5466851

[18] Conte MS, Geraghty PJ, Bradbury AW, Suggested objective performance goals and clinical trial design for evaluating catheter-based treatment of critical limb ischemia. J Vasc Surg. 2009;50(6):1462-1473.e1-3. doi:10.1016/j.jvs.2009.09.044 19897335

[19] Fried LP, Tangen CM, Walston J, ; Cardiovascular Health Study Collaborative Research Group Frailty in older adults: evidence for a phenotype. J Gerontol A Biol Sci Med Sci. 2001;56(3):M146-M156. doi:10.1093/gerona/56.3.M146 11253156

[20] Li G, Thabane L, Ioannidis G, Kennedy C, Papaioannou A, Adachi JD Comparison between frailty index of deficit accumulation and phenotypic model to predict risk of falls: data from the Global Longitudinal Study of Osteoporosis in Women (GLOW) Hamilton cohort. PLoS One. 2015;10(3):e0120144. doi:10.1371/journal.pone.0120144 25764521 PMC4357575

[21] Mijnarends DM, Schols JM, Meijers JM, Instruments to assess sarcopenia and physical frailty in older people living in a community (care) setting: similarities and discrepancies. J Am Med Dir Assoc. 2015;16(4):301-308. doi:10.1016/j.jamda.2014.11.011 25530211

[22] Syddall HE, Westbury LD, Dodds R, Dennison E, Cooper C, Sayer AA Mortality in the Hertfordshire Ageing Study: association with level and loss of hand grip strength in later life. Age Ageing. 2017;46(3):407-412. doi:10.1093/ageing/afw222 27932364 PMC5500162

[23] Rutherford RB, Baker JD, Ernst C, Recommended standards for reports dealing with lower extremity ischemia: revised version. J Vasc Surg. 1997;26:517-538. doi:10.1016/S0741-5214(97)70045-4 9308598

[24] Mills JL Sr., Conte MS, Armstrong DG, The Society for Vascular Surgery Lower Extremity Threatened Limb Classification System: risk stratification based on wound, ischemia, and foot infection (WIfI). J Vasc Surg. 2014;59(1):220-234.e1-2. doi:10.1016/j.jvs.2013.08.003 24126108

[25] Rockwood K, Andrew M, Mitnitski A A comparison of two approaches to measuring frailty in elderly people. J Gerontol A Biol Sci Med Sci. 2007;62(7):738-743. doi:10.1093/gerona/62.7.738 17634321

[26] Arya S, Long CA, Brahmbhatt R, Preoperative frailty increases risk of nonhome discharge after elective vascular surgery in home-dwelling patients. Ann Vasc Surg. 2016;35:19-29. doi:10.1016/j.avsg.2016.01.052 27263810

[27] McRae PJ, Walker PJ, Peel NM, Frailty and geriatric syndromes in vascular surgical ward patients. Ann Vasc Surg. 2016;35:9-18. doi:10.1016/j.avsg.2016.01.033 27238988

[28] Schaller MS, Ramirez JL, Gasper WJ, Zahner GJ, Hills NK, Grenon SM Frailty is associated with an increased risk of major adverse cardiac events in patients with stable claudication. Ann Vasc Surg. 2018;50:38-45. doi:10.1016/j.avsg.2017.12.002 29477684 PMC6014878

[29] Dodds RM, Syddall HE, Cooper R, Kuh D, Cooper C, Sayer AA Global variation in grip strength: a systematic review and meta-analysis of normative data. Age Ageing. 2016;45(2):209-216. doi:10.1093/ageing/afv192 26790455 PMC4776623

[30] Chainani V, Shaharyar S, Dave K, Objective measures of the frailty syndrome (hand grip strength and gait speed) and cardiovascular mortality: a systematic review. Int J Cardiol. 2016;215:487-493. doi:10.1016/j.ijcard.2016.04.068 27131770

[31] Leong DP, Teo KK, Rangarajan S, ; Prospective Urban Rural Epidemiology (PURE) Study investigators Prognostic value of grip strength: findings from the Prospective Urban Rural Epidemiology (PURE) Study. Lancet. 2015;386(9990):266-273. doi:10.1016/S0140-6736(14)62000-6 25982160

[32] Dudzińska-Griszek J, Szuster K, Szewieczek J Grip strength as a frailty diagnostic component in geriatric inpatients. Clin Interv Aging. 2017;12:1151-1157. doi:10.2147/CIA.S140192 28794619 PMC5538538

[33] Adam DJ, Beard JD, Cleveland T, ; BASIL trial participants Bypass versus angioplasty in severe ischaemia of the leg (BASIL): multicentre, randomised controlled trial. Lancet. 2005;366(9501):1925-1934. doi:10.1016/S0140-6736(05)67704-5 16325694

[34] Mehaffey JH, Hawkins RB, Fashandi A, Lower extremity bypass for critical limb ischemia decreases major adverse limb events with equivalent cardiac risk compared with endovascular intervention. J Vasc Surg. 2017;66(4):1109-1116.e1. doi:10.1016/j.jvs.2017.04.036 28655549 PMC5612844

[35] van Haelst STW, Koopman C, den Ruijter HM, Cardiovascular and all-cause mortality in patients with intermittent claudication and critical limb ischaemia. Br J Surg. 2018;105(3):252-261. doi:10.1002/bjs.10657 29116654

[36] Baubeta Fridh E, Andersson M, Thuresson M, Amputation rates, mortality, and pre-operative comorbidities in patients revascularised for intermittent claudication or critical limb ischaemia: a population based study. Eur J Vasc Endovasc Surg. 2017;54(4):480-486. doi:10.1016/j.ejvs.2017.07.005 28797662

[37] Montero-Baker M, Zulbaran-Rojas A, Chung J, Endovascular therapy in an “all-comers” risk group for chronic limb-threatening ischemia demonstrates safety and efficacy when compared with the established performance criteria proposed by the Society for Vascular Surgery. Ann Vasc Surg. 2020;67:425-436. doi:10.1016/j.avsg.2020.03.008 32209405

[38] Takeji Y, Yamaji K, Tomoi Y, Impact of frailty on clinical outcomes in patients with critical limb ischemia. Circ Cardiovasc Interv. 2018;11(7):e006778. doi:10.1161/CIRCINTERVENTIONS.118.006778 30006333

[39] van Aalst FM, Verwijmeren L, van Dongen EPA, Frailty and functional outcomes after open and endovascular procedures for patients with peripheral arterial disease: a systematic review. J Vasc Surg. 2020;71(1):297-306.e1. doi:10.1016/j.jvs.2018.12.060 31331651

[40] Alvarez-Nebreda ML, Bentov N, Urman RD, Recommendations for preoperative management of frailty from the Society for Perioperative Assessment and Quality Improvement (SPAQI). J Clin Anesth. 2018;47:33-42. doi:10.1016/j.jclinane.2018.02.011 29550619

